# Editorial: Ovarian aging: pathophysiology and recent development of maintaining ovarian reserve, volume III

**DOI:** 10.3389/fendo.2024.1460934

**Published:** 2024-08-05

**Authors:** Osamu Hiraike, Akira Iwase, Antonio Simone Laganà

**Affiliations:** ^1^ Department of Obstetrics and Gynecology, Faculty of Medicine, The University of Tokyo, Tokyo, Japan; ^2^ Department of Obstetrics and Gynecology, Gunma University Graduate School of Medicine, Maebashi, Japan; ^3^ Unit of Obstetrics and Gynecology, “Paolo Giaccone” Hospital, Department of Health Promotion, Mother and Child Care, Internal Medicine and Medical Specialties (PROMISE), University of Palermo, Palermo, Italy

**Keywords:** ovary, ageing, artificial reproductive technologies, ovarian follicle, estrogen

The function of the ovary must be considered both quantitatively and qualitatively. It is important to know the number of remaining of oocytes in the ovary, and it is also important to know whether the oocyte can become a good embryo after fertilization for the establishment of conception. The oocyte, granulosa cells, and theca cells form a follicle, and these components are indispensable for the development of mature oocytes. For the oocyte to function properly, it must undergo an elaborate process of ovarian development and oocyte formation, utilizing the central and local control mechanisms of ovulation and follicular development, luteinization, and follicle atresia, which is important in human reproduction. The development of experimental methods to predict ovarian dysfunction is an important issue in the management of reproductive-aged women and women after menopause. Establishment of appropriate diagnostic and therapeutic methods remains a major challenge.

Against this background, “Ovarian Aging: Pathophysiology and Recent Development of Maintaining Ovarian Reserve” was first proposed in 2019. Since then, the III volume was introduced. A further volume reflects the interests of researchers in this area, proving it remains a fascinating issue. In assisted reproductive technologies (ART), a number of new technologies have been introduced including cryopreservation of eggs and ovarian tissue. A recent trend of using next-generation sequencing technologies in the clinical field has provided us important insights and analysis of preimplantation embryos and bacterial flora has become a daily setting. The study of follicle development was previously impossible, but recent progress in molecular biology significantly improved our understanding of the differentiation of oocytes and the microenvironment of ovarian follicles.

We aimed to overview the recent studies related to ovarian function, including follicular pathophysiology, development of ovarian markers, drugs that maintain or improve ovarian reserve, and technologies of cryopreservation of oocyte and ovarian tissues. With the aim of this Research Topic, nine groups responded to our recruitment and contributed and gave us useful insights into the underlying mechanisms of ovarian aging. The results shown here are interesting and we are confident that these studies offer a comprehensive collection on ovarian aging and its related issues. Fortunately, this third volume of Ovarian Ageing resulted in 25,207 total views, 8,148 article views, 3,024 downloads, and 17,059 topic views (accessed on 29 Jun, 2024).

In this series, three researchers provided clinical suggestions on ART. The clinical study [He et al.] showed that the ratio of basal FSH/LH could predict ovarian response and they developed an ovarian sensitivity index that can be used as an indicator of ovarian response in ART treatment. Sun et al. showed the progesterone to number of mature oocytes index levels can be useful for predicting pregnancy outcome in fresh IVF/ICSI cycles. A clinical randomized trial [Chu et al.] has proven beneficial for women with decreased ovarian reserve because it showed that intake of flaxseed oil contributed to the formation of MII oocytes. The manuscript using a machine learning model [Ding et al.] clearly demonstrated that the representative ovarian reserve marker, anti-Müllerian hormone (AMH), and antral follicle count (AFC) were applicable to a group of women aged 20 to 35 years.

Since the discovery of estrogen receptor α (ERα), its function in the ovary was investigated because estrogens play crucial roles in the development of ovarian follicles. It was known that ovaries of mice deficient in ERα exhibit multiple hemorrhagic cysts in the ovaries, but the physiological role of the cyst formation was not known. Schröder et al. showed iron deposits in the ovaries and significant increases in ovarian mast cells involved in iron-mediated foam cell formation, resembling signs of aging [Luo et al.].

Vasomotor symptoms such as hot flashes and sweating are representative climacteric symptoms and can be reversed by the supplementation of estrogens, known as hormonal therapy (HT). However, we sometimes encounter postmenopausal patients with vasomotor symptoms that are resistant to the conventional HT. A clinical trial [Li et al.] showed the beneficial effects of stellate ganglion block on perimenopausal hot flashes, and we can apply the result of this study in daily clinical settings.

The review manuscripts shed light on the role of mitochondria in ovarian aging [Ju et al.] and the usefulness of acupuncture, which is known as a less invasive technique and can be applied to various diseases [Cao et al.].

## Concluding remarks

Altogether, the clinical studies, research studies, and reviews contained in this topic are insightful and beneficial both for clinicians and researchers. The wide range of data provides a good overview of the topic, and we are sure that these novel studies might enhance future studies with the aim to improve and maximize infertility treatment and the medical care of postmenopausal women.

## Author contributions

OH: Conceptualization, Data curation, Formal analysis, Funding acquisition, Investigation, Methodology, Project administration, Resources, Software, Supervision, Validation, Visualization, Writing – original draft, Writing – review & editing. AI: Writing – review & editing. AL: Writing – review & editing.

